# Future worldwide coronavirus disease 2019 epidemic predictions by Gaidai multivariate risk evaluation method

**DOI:** 10.1002/ansa.202400027

**Published:** 2024-08-27

**Authors:** Oleg Gaidai, Yu Cao, Yan Zhu, Alia Ashraf, Zirui Liu, Hongchen Li

**Affiliations:** ^1^ Department of Mechanics and Mathematics Ivan Franko Lviv State University Lviv Ukraine; ^2^ College of Engineering Science and Technology Shanghai Ocean University Shanghai China; ^3^ School of Naval Architecture and Ocean Engineering Jiangsu University of Science and Technology Zhenjiang China

**Keywords:** artificial intelligence, COVID‐19, epidemic outbreak, mathematical biology, public health

## Abstract

Accurate estimation of pandemic likelihood in every US state of interest and at any time. Coronavirus disease 2019 (COVID‐19) is an infectious illness with a high potential for global dissemination and low rates of fatality and morbidity, placing some strains on national public health systems. This research intends to benchmark a novel technique, that enables hazard assessment, based on available clinical data, and dynamically observed patient numbers while taking into account pertinent territorial and temporal mapping. Multicentre, population‐based, and biostatistical strategies have been utilized to process raw/unfiltered medical survey data. The expansion of extreme value statistics from the univariate to the bivariate situation meets with numerous challenges. First, the univariate extreme value types theorem cannot be directly extended to the bivariate (2D) case,—not to mention challenges with system dimensionality higher than 2D. Assessing outbreak risks of future outbreaks in any nation/region of interest. Existing bio‐statistical approaches do not always have the benefits of effectively handling large regional dimensionality and cross‐correlation between various regional observations. These methods deal with temporal observations of multi‐regional phenomena. Apply contemporary, novel statistical/reliability techniques directly to raw/unfiltered clinical data. The current study outlines a novel bio‐system hazard assessment technique that is particularly suited for multi‐regional environmental, bio, and public health systems, observed over a representative period. With the use of the Gaidai multivariate hazard assessment approach, epidemic outbreak spatiotemporal risks may be properly assessed. Based on raw/unfiltered clinical survey data, the Gaidai multivariate hazard assessment approach may be applied to a variety of public health applications. The study's primary finding was an assessment of the risks of epidemic outbreaks, along with a matching confidence range. Future global COVID‐19/severe acute respiratory syndrome coronavirus 2 (SARS‐COV2) epidemic risks have been examined in the current study; however, COVID‐19/SARS‐COV2 infection transmission mechanisms have not been discussed.

**Abbreviations**
AI: artificial intelligence
COVID‐19: coronavirus disease 2019
CI: confidence interval
SARS‐COV2: severe acute respiratory syndrome coronavirus 2
EVT: Extreme Value Theory
MDOF: multi degree of freedom (system)
MUR: Mean Up‐crossing Rate
NAG: Numerical Algorithm Group
SQP: Sequential Quadratic Programming
PDF: Probability Density Function
SODP: 2^nd^ Order Difference Plot

## 1 INTRODUCTION

**FIGURE 1:**
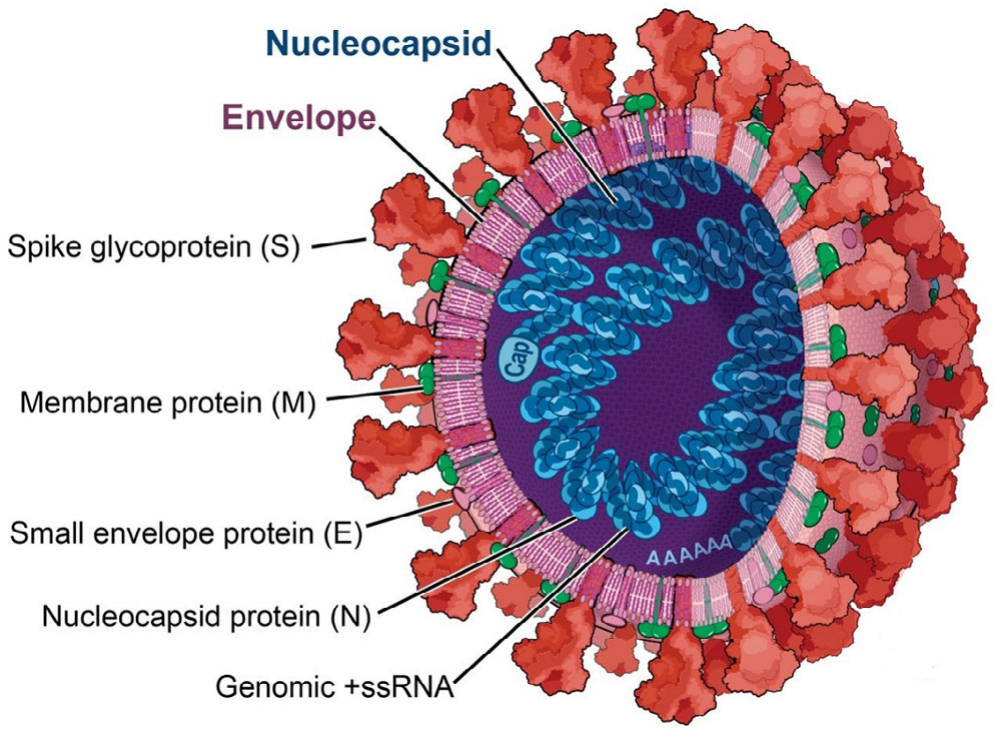
Coronavirus schematic image, https://www.ccjm.org/content/87/6/321/tab‐figures‐data.

Coronavirus disease 2019/severe acute respiratory syndrome coronavirus 2 (COVID‐19/SARS‐COV2) and other recent outbreaks statistics, having comparable epidemiologic patterns, had drawn significant research interest,^1^, ^2^, ^3^, ^4^, ^5^, ^6^, ^7^, ^8^, ^9^, ^10^ Figure 1. In general, using conventional theoretical statistical multivariate methods, given actual epidemic settings, is relatively challenging to assess outbreak risks affected by ambient biological covariates.^11^, ^12^, ^13^, ^14^, ^15^, ^16^, ^17^, ^18^ In principle, direct MC (i.e., Monte Carlo) numerical simulations or a sufficient number of clinical observations might be used to assess the reliability of complex biological systems. This study has been focused on global COVID‐19/SARS‐COV2 outbreaks cross‐correlations across different nations/countries.^19^, ^20^, ^21^, ^22^, ^23^, ^24^, ^25^, ^26^ Further studies on statistical variances per nation have been provided.^27^ COVID‐19/SARS‐COV2's clinical raw/unfiltered data, along with associated research, is publicly accessible.^27^, ^28^, ^29^, ^30^, ^31^, ^32^, ^33^, ^34^, ^35^

Authors utilized EVT (i.e., Extreme Value Theory), to assess the risks of a worldwide influenza pandemic, suggesting prospective projections for epidemic risks.^9^ Similarly,^10^ utilized EVT to predict and identify flu epidemic abnormalities. Due to a lack of statistical research to forecast multivariate risks of influenza and infectious disease outbreaks, the authors suggested a novel multivariate approach, providing improved insight and predictors. Given that epidemic outbreaks are viewed as unanticipated events, that might occur at any time and in any region/country, the current study employs a spatiotemporal approach. To forecast pandemic risks, wherever they may occur, specific non‐dimensional factors have been introduced.^36^, ^37^, ^38^, ^39^, ^40^, ^41^, ^42^, ^43^ Environmental effects/covariates. acting on biological systems follow cyclical/seasonal patterns. The alternative is to view an epidemiological process, as being reliant on several external bio‐covariates, whose temporal variation may be described as quasi‐ergodic processes. Clinical data for COVID‐19/SARS‐COV2 in 195 countries from February 2020 to the end of 2022 had been retrieved from a public source.^1^ The global biological system under investigation may be modeled as an MDOF (i.e., multi‐degree of freedom) bio‐dynamic system, with strongly inter‐correlated regional/national key/critical components/dimensions. Several recent researchers have previously utilized advanced statistical techniques, to forecast COVID‐19/SARS‐COV2 progression; for linear log models.^21^

**FIGURE 2:**
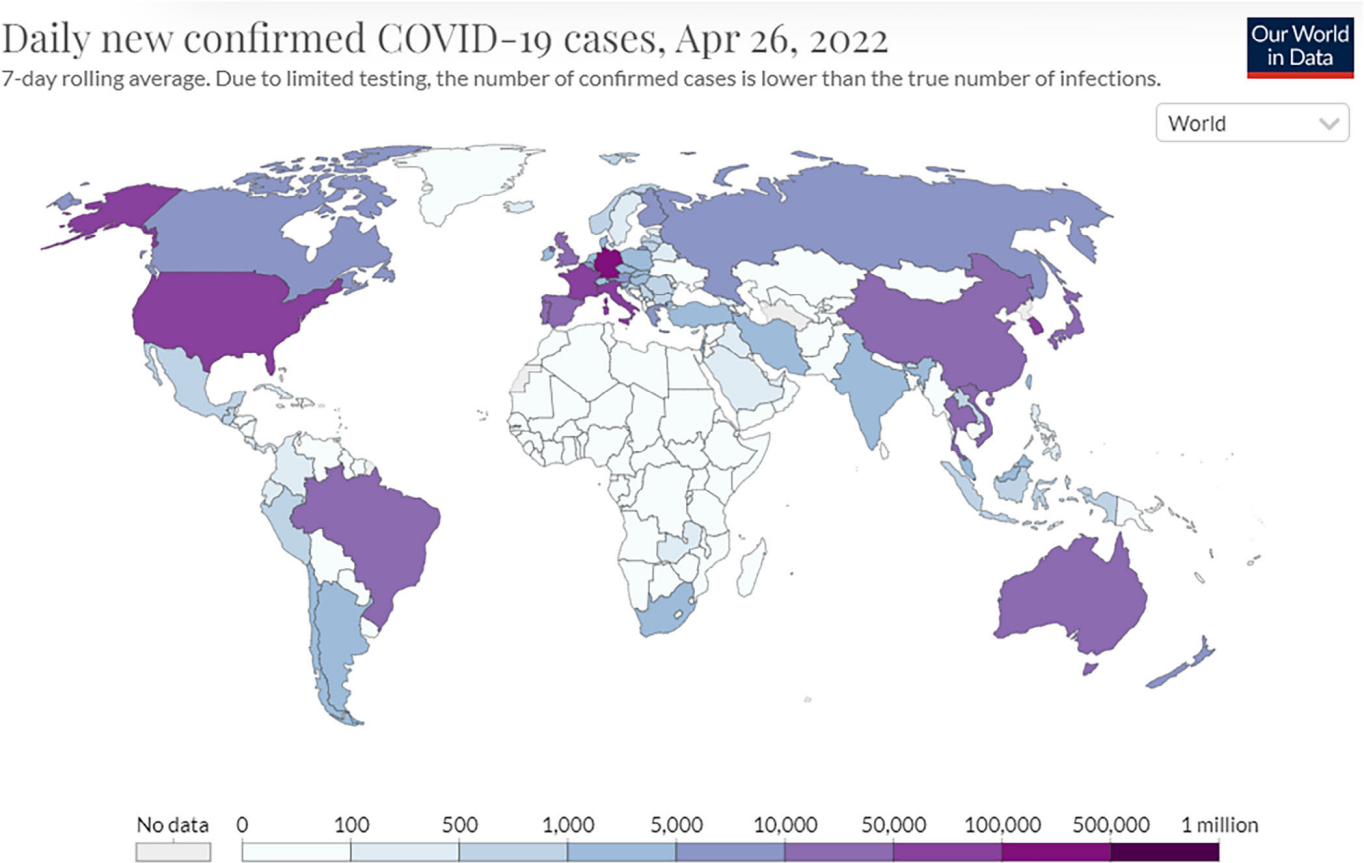
Map of the world's countries with recorded coronavirus disease 2019/severe acute respiratory syndrome coronavirus 2 (COVID‐19/SARS‐COV2) cases, 1.

Although the main goal of this study was to predict future epidemic outbreak risks, this study solely concentrated on daily numbers of newly reported cases, ignoring symptoms themselves. For detailed information related to so‐called long‐lasting COVID‐19/SARS‐COV2 symptoms, or the “long COVID”. For more details on mortality study see.^12^, ^14^ A global map with COVID‐19/SARS‐COV2 cases is given in Figure 2.

## 2 GAIDAI MULTIVARIATE RISKS EVALUATION METHOD

The underlying epidemiological process has been considered in this analysis to be seasonally variable, but statistically representative across 2 (2020–2022) years of continuous clinical observation. This study was based on the quasi‐stationarity assumption. The problem of the underlying tendency/trend has to be addressed given a longer time horizon, for example, 10–100 years.^8^ Novel multivariate risks evaluation methodology,^7^ to be briefly described, that is particularly suited for temporally and geographically non‐homogeneous biological and public health systems.^44^, ^45^, ^46^, ^47^, ^48^

**FIGURE 3:**
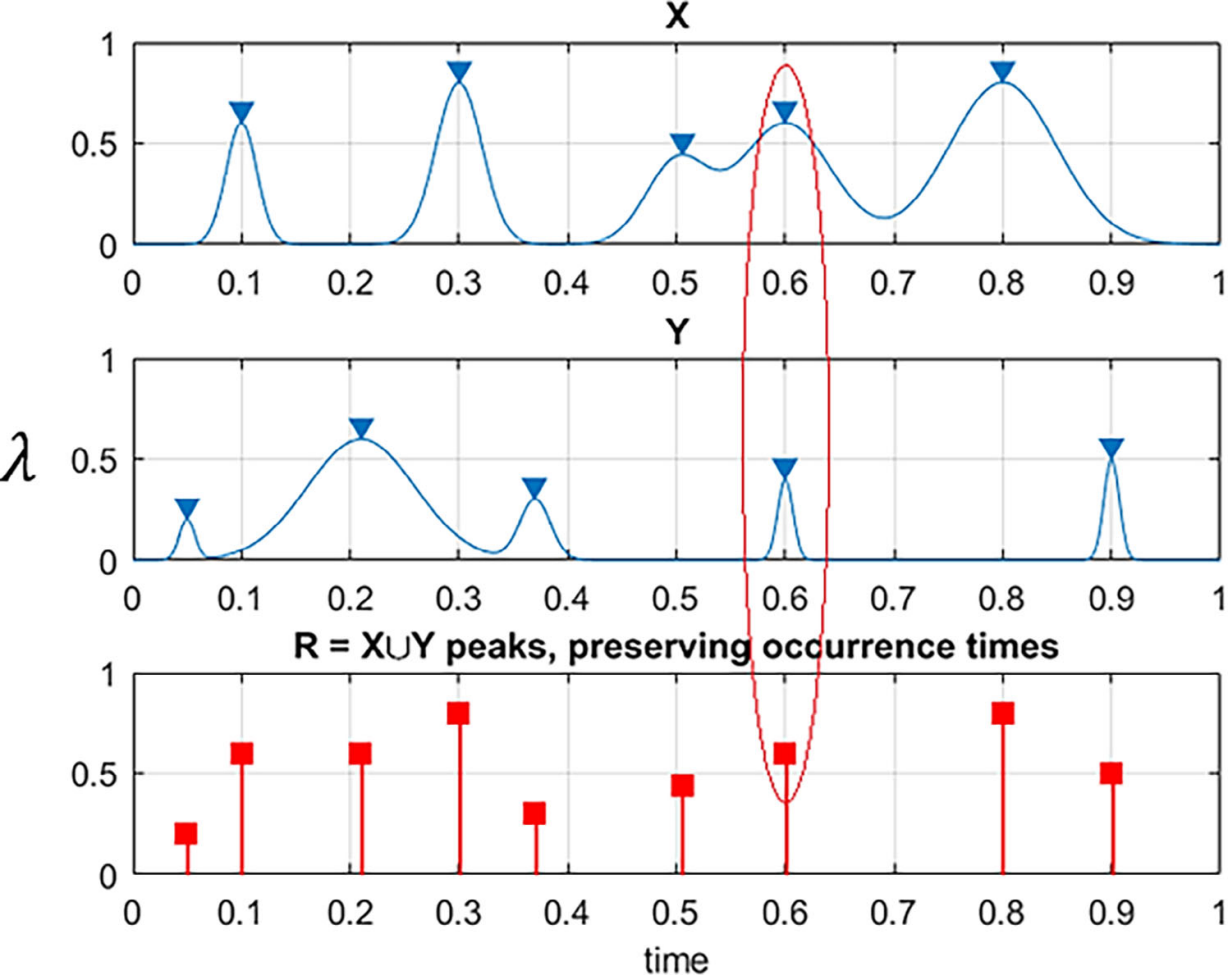
Example of how two key components, X and Y, are coalesced into 1 new synthetic vector $\vec{R}$. Red ellipse marking simultaneous maxima for two different system's bio‐components, $\lambda_{\mathrm{cr}}=1$.

Considering piecewise jointly‐stationary, MDOF environmental public health dynamic biosystem, having its key/critical components X(t),Y(t),Z(t),… being part of biosystem's dynamic MDOF time‐record (X(t),Y(t),Z(t),…), observed/recorded/measured over sufficient (i.e., representative) timelapse (0,T). 1D system's critical component's global maxima are denoted here as $X_{T}^{\max}=\max_{0\leq t\leq T}X\left(t\right)$, $Y_{T}^{\max}=\max_{0\leq t\leq T}Y\left(t\right)$, $Z_{T}^{\max}=\max_{0\leq t\leq T}Z\left(t\right),\;\text{…}$ covering the whole timelapse (0,T). By suitably long (representative) timelapse T, one essentially means a large enough value of T concerning dynamic public health biosystem's auto‐correlation and relaxation times.^49^, ^50^, ^51^, ^52^, ^53^, ^54^ Let $X_{1},\text{…},X_{N_{X}}$ be environmental public health system key/critical component's local maxima of the critical component process X(t) at discrete instants of time‐instants, temporally increasing, $t_{1}^{X}<\cdots <t_{N_{X}}^{X}$ detected within (0,T). Definitions for the remaining MDOF dynamic public health environmental system's key/critical components, Y(t),Z(t),… with $Y_{1},\text{…},Y_{N_{Y}};$ $Z_{1},\text{…},Z_{N_{Z}}$ etc., being quite similar. It had been assumed that all environmental public health biosystems' critical components' local maxima are non‐negative. The goal was to accurately determine risks of dynamic environmental public health biosystem's hazard/failure risk/probability

(1) $$P_{F}=\mathrm{Prob}\left(X_{T}^{\max}>\eta_{X}\;\cup \;Y_{T}^{\max}>\eta_{Y}\;\cup \;Z_{T}^{\max}>\eta_{Z}\;\cup \;\text{…}\right)$$

related to the target biosystem's survival chances/probability P, being expressed as $P\equiv 1-P_{F}=\;\int \int \;\int_{0,\;0,\;0,\;\text{…}}^{\eta_{X},\;\eta_{Y},\;\eta_{Z},\;\text{…}}p_{X_{T}^{\max},Y_{T}^{\max},\;Z_{T}^{\max},\;\text{…}}\;\left(x_{T}^{\max},\;y_{T}^{\max},\;z_{T}^{\max},\;\text{…}\right)\;dx_{T}^{\max}dy_{T}^{\max}dz_{T}^{\max}\text{…}$ being the target public health biosystem's probability of non‐exceedance of all public health biosystem's principal component's values $\eta_{X}$, $\eta_{Y}$, $\eta_{Z}$,… simultaneously; with ∪ standing for logical unity operator; and $p_{X_{T}^{\max},\;Y_{T}^{\max},\;Z_{T}^{\max},\;\text{…}}$ being target joint PDF of principal component's global maxima, over observational timelapse (0,T). Next, MDOF public health system's vector (X(t),Y(t),Z(t),…) to be scaled to the nondimensional version: $\left(\tilde{X},\tilde{Y},\;\tilde{Z},\;\text{…}\right)$, with $\tilde{X}=\frac{X}{\eta_{X}},\;\tilde{Y}=\frac{Y}{\eta_{Y}},\;\tilde{Z}=\frac{Z}{\eta_{Z}},$…. It is not practicable to straightforwardly assess the latter public health system's joint PDF, due to the public health biosystem's multi‐dimensionality, along with given limitations of the underlying raw clinical dataset. More specifically, the environmental dynamic system is considered to have failed/damaged, or entered into a state of hazard, when either biosystem's critical components X(t) exceeds $\eta_{X}$; or Y(t) exceeds $\eta_{Y}$; or Z(t) exceeds $\eta_{Z}$, etc.,—or, equivalently, when either $\tilde{X},\tilde{Y},\;\tilde{Z},\;\text{…}$ exceeds 1. Let one arrange the public health system's key component's local maxima temporal‐instants $\left[t_{1}^{X}<\cdots <t_{N_{X}}^{X};\;t_{1}^{Y}<\cdots <t_{N_{Y}}^{Y};\;t_{1}^{Z}<\cdots <t_{N_{Z}}^{Z}\right]$ into a single temporal merged bio‐systems vector, $t_{1}\leq \cdots \leq t_{N}$, in a monotonously non‐decreasing temporal order, having $t_{N}=\max\;\left\{t_{N_{X}}^{X},\;t_{N_{Y}}^{Y},\;t_{N_{Z}}^{Z},\;\text{…}\right\}$, $N\leq N_{X}+N_{Y}+\;N_{Z}+\;\text{…}$ Local maxima of each of MDOF environmental biosystem's load/response principal components, namely X(t) or Y(t), or Z(t), etc., being represented by their occurrence times $t_{j}$. 1D environmental biosystem principal components $\left(\tilde{X},\tilde{Y},\;\tilde{Z},\;\text{…}\right)$ local maxima being combined/coalesced, coherent with merged/coalesced temporal vector $t_{1}\leq \cdots \leq t_{N}$, forming temporally increasing synthetic nondimensional public health biosystem's vector $R\left(t\right)\equiv \;\vec{R}=\left(R_{1},\;R_{2},\;\text{…},R_{N}\right)$, with $\;R_{j}=\;\max\;\left\{\left({\tilde{X}}_{j}|\;\exists \;j_{X},\;t_{j_{X}}^{X}=t_{j}\;\right),\;\left({\tilde{Y}}_{j}|\;\exists \;j_{Y},\;t_{j_{Y}}^{Y}=t_{j}\;\right),\;\left({\tilde{Z}}_{j}|\;\exists \;j_{Z},\;t_{j_{Z}}^{Z}=t_{j}\;\right),\;\text{…}\right\}$ for j=1,…,N, see Figure 3. Next, the “scaling” parameter 0<λ≤1 is introduced, to artificially reduce hazard/limit/risk values for all bio‐systems nondimensionalized principal components. Public health system's survival chances/probability P(λ) being defined as smooth $C^{1}$ function of scaling parameter λ; with P≡P(1) according to Equation (1). Estimation of the survival (non‐exceedance) probability P(λ) now being as follows:

(2) $$\begin{array}{ccc} P\left(\lambda \right) & = & \mathrm{Prob}\left\{R_{N}\leq \lambda ,\text{…},R_{1}\leq \lambda \right\}\\ & = & \mathrm{Prob}\left\{R_{N}\leq \lambda \;|\;R_{N-1}\leq \lambda ,\text{…},R_{1}\leq \lambda \right\}\cdot \mathrm{Prob}\left\{R_{N-1}\leq \lambda ,\text{…},R_{1}\leq \lambda \right\}\\ & = & \prod_{j=2}^{N}P\;\mathrm{rob}\left\{R_{j}\leq \lambda \;|\;R_{j-1}\leq \lambda ,\text{…},R_{1}\leq \lambda \right\}\cdot \mathrm{Prob}\left(R_{1}\leq \lambda \right) \end{array}$$

To account for dependencies between neighboring $R_{j}$, a 1‐step public health biosystem's memory approximation (conditioning memory depth number k=1) being implemented

(3) $$\mathrm{Prob}\left\{R_{j}\leq \lambda \;|\;R_{j-1}\leq \lambda ,\text{…},R_{1}\leq \lambda \right\}\approx \mathrm{Prob}\left\{R_{j}\leq \lambda \;|\;R_{j-1}\leq \lambda \right\}$$

for 2≤j≤N with conditioning memory depth number k=2. Equation (3) may be now expressed as

(4) $$\mathrm{Prob}\left\{R_{j}\leq \lambda \;|\;R_{j-1}\leq \lambda ,\text{…},R_{1}\leq \lambda \right\}\approx \mathrm{Prob}\left\{R_{j}\leq \lambda \;|\;R_{j-1}\leq \lambda ,\;R_{j-2}\leq \lambda \right\}$$

with 3≤j≤N (conditioning memory depth number k=3), etc. By tracking each hazard/failure/risk/damage event, happening sequentially in time, the intention is now to prevent cascading/clustering public health biosystem's inter‐correlated exceedances. Since MDOF dynamic environmental process R(t) had been considered to be piecewise ergodic, thus quasi‐stationary, probability/risk $p_{k}\left(\lambda \right)≔Prob\{R_{j}>\lambda \;|\;R_{j-1}\leq \lambda ,\;\text{…},\;R_{j-k+1}\leq \lambda \}\;\mathrm{for}\;j\geq k$ will be also independent of j and solely dependent on conditioning memory depth number k. Thus, non‐exceedance (i.e., survival) probability/chances may be approximately assessed, using Poisson's assumption^55^, ^56^, ^57^, ^58^, ^59^, ^60^:

(5) $$P_{k}\left(\lambda \right)\approx \exp\;\left(-N\cdot p_{k}\left(\lambda \right)\right)\;,\;k=1,\;2,\;3,\text{…}$$

$\mathrm{Prob}(R_{1}\leq \lambda )\approx 1$ being ignored in Equation (5) since the chance of the design public health biosystem's failure/hazard/damage probability/risk must be of a low order of magnitude, along with N≫k.^61^, ^62^, ^63^, ^64^, ^65^

Poisson‐type PDF, given by $f_{\mathrm{Poisson}}\left(x\right)=e^{-\lambda_{P}}\;\lambda_{P}^{x}/x!$ with $\lambda_{P}$ being Poisson parameter can be modified following Equation (5) with parameter $p\left(\lambda \right)N/T\approx \nu^{+}\left(\lambda \right)\cdot \;T$, accounting for clustering, instead of assuming independent temporally successive up‐crossing events of the high/critical λ levels (in the current study λ≥1) can be made. Regarding conditioning memory depth number k, there is a natural way of convergence

(6) $$P=\lim_{k\to \infty }P_{k}\left(\lambda_{cr}\right);\;p\;\left(\lambda \right)=\lim_{k\to \infty }p_{k}\left(\lambda \right)$$

with $\lambda_{cr}$ being the target critical level/threshold. Note that Equation (5) for k=1 turns into well‐known non‐exceedance probability relationship with the corresponding MUR (Mean Up‐crossing Rate) function

(7) $$P\left(\lambda \right)\;\approx \exp\;\left(-\nu^{+}\left(\lambda \right)\;T\right);\;\nu^{+}\left(\lambda \right)=\int_{0}^{\infty }\zeta p_{R\dot{R}}\left(\lambda ,\zeta \right)d\zeta$$

with $\nu^{+}\left(\lambda \right)$ being MUR of dynamic response level λ for the non‐dimensional public health biosystem's vector R(t), introduced above. The Rice's formula, given by Equation (7), yields MUR. Figure 3 shows that the constructed synthetic $\vec{R}$‐vector has net 0 data loss.^66^, ^67^, ^68^, ^69^, ^70^, ^71^

Rice's formula is found in Equation (7), where $\dot{R}$ being the time derivative $R^{\prime }\left(t\right)$, and $p_{R\dot{R}}$ being the joint PDF (Probability Density Function) for $\left(R,\;\dot{R}\right)$. For further detailed information.^72^, ^73^, ^74^, ^75^, ^76^, ^77^, ^78^ Equation (7) relies on Poisson's assumption that separate up‐crossing occurrences corresponding to high λ levels (in this article, $\lambda \geq \lambda_{cr}$) may be assumed nearly independent. Due to intrinsic interdependencies between bio‐components temporally neighbouring out‐crossings, the latter may not be true for narrowband bio‐processes exhibiting cascading hazards/failures in different dimensions, temporally one after the other. Given the constructed synthetic vector $\vec{R}$, these interdependencies form clusters/groups of strongly connected biosystem principal component's local maxima $R\left(t\right)\;\equiv \;\vec{R}=\left(R_{1},\;R_{2},\;\text{…},R_{N}\right)$. As already mentioned, the bio‐system's joint quasi‐stationarity premise has been used. However, Gaidai risks evaluation methodology may be well utilized for nonstationary cases.^78^, ^79^, ^80^, ^81^, ^82^, ^83^, ^84^, ^85^, ^86^, ^87^, ^88^, ^89^ Given the probability of each transient seasonal bio‐condition of $q_{m}$ within the nonstationary case's scatter diagram of m=1,…,M one has $\sum_{m\;=\;1}^{M}q_{m}=1$. Next,

(8) $$p_{k}\left(\lambda \right)\equiv \sum_{m\;=\;1}^{M}p_{k}\left(\lambda ,m\right)q_{m}$$

being the same functions as in Equation (6), with the exception that $p_{k}\left(\lambda ,m\right)$ corresponds now to a particular short‐term seasonal epidemic condition, with the number m. The $p_{k}\left(\lambda \right)$ functions mentioned above are frequently regular in the PDF tail, especially for values λ of approaching, and surpassing 1. The NAG (i.e., Numerical Algorithm Group) Library's SQP (i.e., Sequential Quadratic Programming) approach may also be used to assess 4‐parameter Weibull‐type PDF parameters.^79^, ^80^, ^81^, ^82^, ^83^, ^84^, ^85^

95% CIs (i.e., confidence intervals) $\left({\mathrm{CI}}^{-}\left(\lambda \right),\;{\mathrm{CI}}^{+}\left(\lambda \right)\right)$, may be estimated empirically from the underlying raw/unfiltered MC simulated/clinically measured dataset. For target levels of λ reaching 1, *p‐*% CI of $p_{k}\left(\lambda \right)$ maybe directly represented as

(9) $${\mathrm{CI}}^{\pm }\left(\lambda \right)=p_{k}\;\left(\lambda \right)\left(1\pm \frac{f\left(p\right)}{\sqrt{\left(N-k+1\right)p_{k}\left(\lambda \right)}}\right)$$

having f(p) obtained from the inverse Gaussian‐type PDF, having N as a total number of discrete underlying data points.

## 3 EXPERIMENT

Making predictions, regarding influenza‐like epidemics has long been a focus of epidemiology as well as mathematical biology. Dynamics of public health systems are widely acknowledged to constitute highly nonlinear dynamic systems, spanning over numerous dimensions, yet being spatially cross‐correlated. Earlier studies have used a variety of techniques to model the dynamics of influenza‐like diseases. The current section illustrates the efficacy of the Gaidai multivariate risks evaluation methodology, described above using a novel strategy, applied to clinical COVID‐19/SARS‐COV2 datasets, consisting of daily recorded infected patient time‐series, spread over wide terrains. Both COVID‐19/SARS‐COV2 and influenza are contagious illnesses, that have spread throughout the world with low morbidity and mortality. Seasonally in the Northern Hemisphere, they occur most commonly in late fall, winter, and early spring, with the winter season seeing their peak occurrence. WHO (i.e., World Health Organization) estimates that every year, seasonal influenza‐kind epidemics brought on by the influenza A and B virus types cause between 0.3% and 0.4% of cases of severe illness and between 0.03% and 0.06% of deaths worldwide, placing some burdens on global public health.^14^ Numbers in this section have been sourced from the World in Data website, 1; this website provided daily global total COVID‐19/SARS‐COV2 cases that were diagnosed from 22 January 2020 to the end of 2022. Key/critical bio‐components X,Y,Z,… have been selected equal to new daily reported patient numbers across 195 distinct countries/nations, creating an example of a 195‐dimensional (i.e., 195D) dynamic biological public health system. Next, 195 measured time series X,Y,Z,…. had been scaled as follows

(10) $$X\to \frac{X}{\eta_{X}},\;Y\to \frac{Y}{\eta_{Y}},\;Z\to \frac{Z}{\eta_{Z}},\;\text{…}$$

Thus, making 195 biosystem principal components non‐dimensional, having identical hazard/failure/damage thresholds/limits $\lambda_{cr}$. Hazard/failure/risk/damage limits/thresholds $\eta_{X},\;\eta_{Y},\;\eta_{Z},\;\text{…}$, or epidemic thresholds, being not straightforward to assess. The easiest option would be for various nations to establish risk/hazard/failure/damage limits equal to the population of the associated nation/country, as a percentage (%) of the local population, thereby making X,Y,Z,… equal to the proportion of daily infected people, per country. The next step is now to combine all local maxima from 195 recorded time series to form a single synthetic time series while maintaining their temporal non‐decreasing order: $\vec{R}=\left(\max\left\{X_{1},Y_{1},Z_{1},\text{…}\right\},\text{…},\max\left\{X_{N},Y_{N},Z_{N},\text{…}\right\}\right)$.^86^, ^87^, ^88^, ^89^, ^90^, ^91^, ^92^, ^93^

**FIGURE 4:**
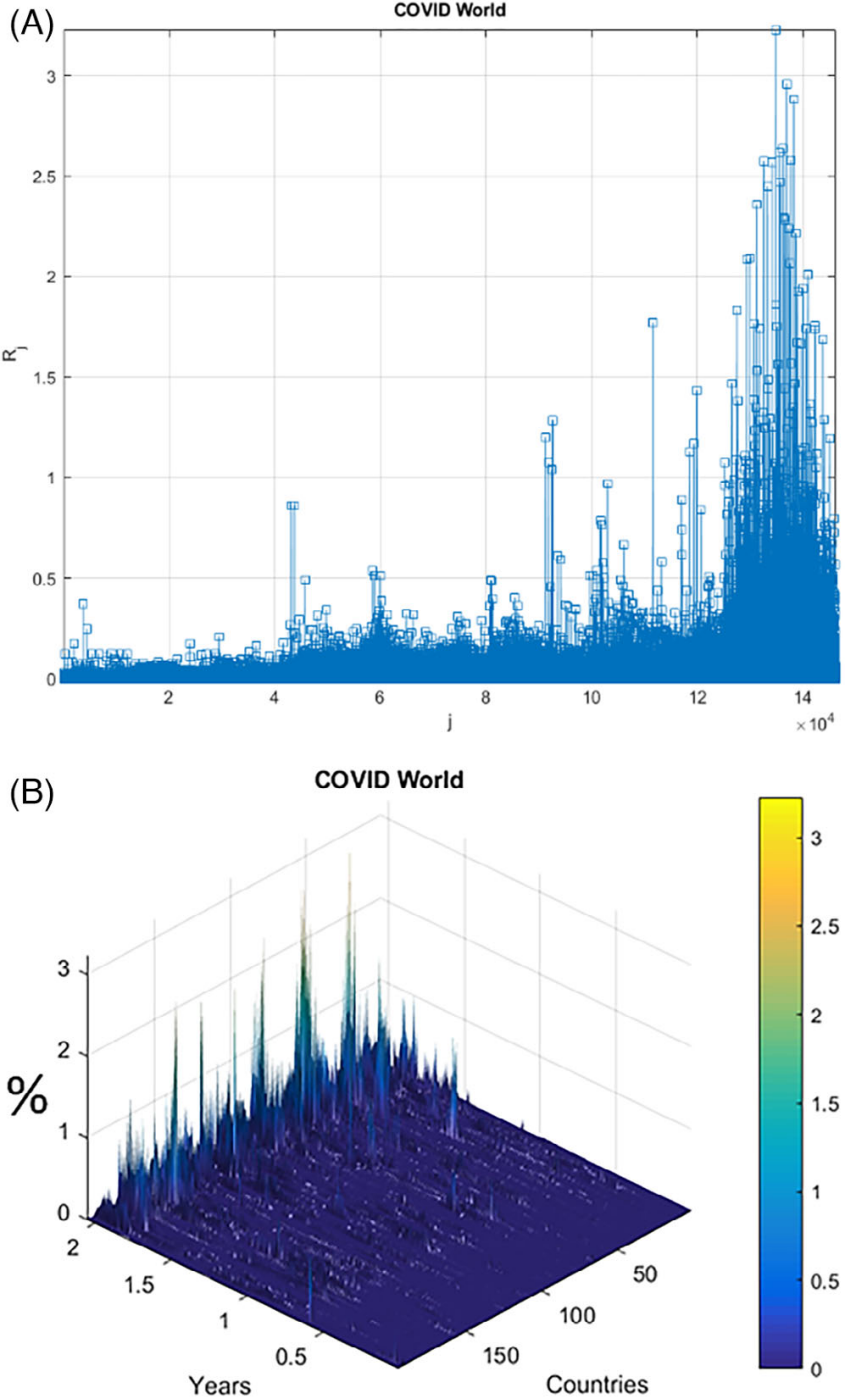
Daily new recorded patient numbers. (A) vector $\vec{R}$, scaled following Equation (9) as % of the corresponding country's population. (B) 2D surface versus time, and each country index.

The number of daily new recorded patients is presented in Figure 4A as a synthetic vector $\vec{R}$ made up of combined regional/national daily new patients, counted per million of the relevant region/country's population.

## 4 RESULTS AND DISCUSSION

Vector $\vec{R}$ is coalesced from various geographical/national principal components having various epidemic backgrounds. Index j is the running index of the vector $\vec{R}$.^94^, ^95^, ^96^, ^97^, ^98^

**FIGURE 5:**
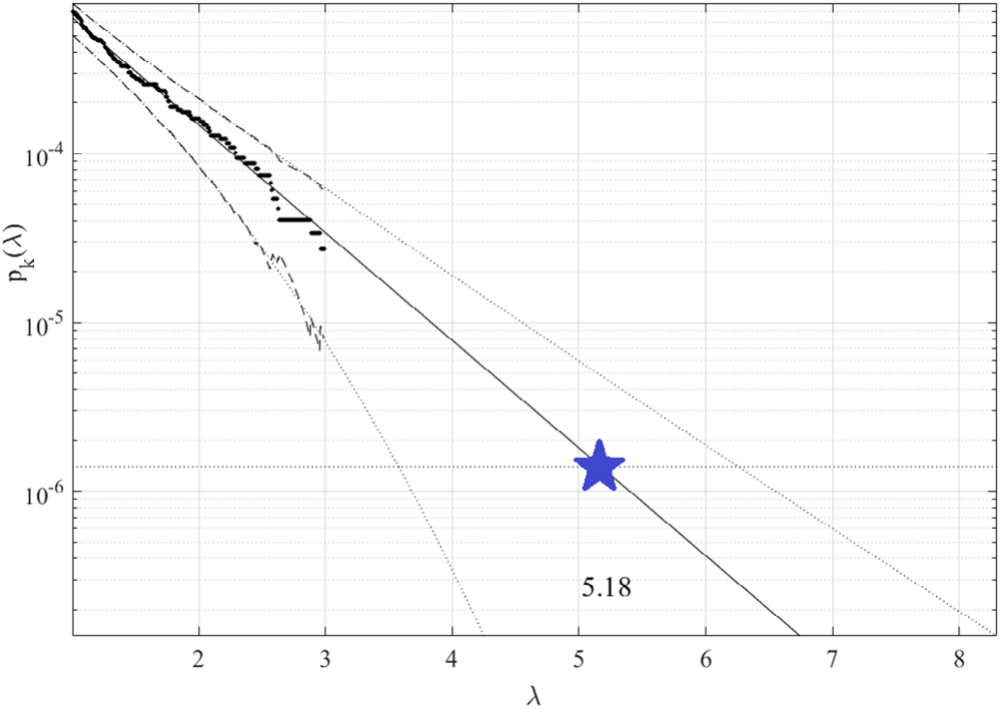
Forecast of daily new patient numbers. The 10‐year return level extrapolation of $p_{k}\left(\lambda \right)$ towards the critical level (star) is a % of the local/national population. Extrapolated 95% CI (i.e., confidence interval) indicated with 2 dotted lines. % of the regional/national population on the x‐axis.

λ=1% cut‐off value had been used on the horizontal x‐axis. Figure 5 shows a forecast of the number of daily new patients, extrapolated following Equation (7), towards epidemic outbreak having a 100‐year return period, indicated with a horizontal dotted line, and somewhat beyond. Extrapolated 95% CI is shown by two dotted lines. The goal hazard/failure probability 1−P from Equation (1) is directly connected to p(λ), following Equation (5). As a result, it is possible to estimate bio‐system hazard/failure/damage probability/risk $1-P\approx 1-P_{k}\left(\lambda_{cr}\right)$ following Equation (5). The total number of local maxima within unified synthetic vector $\vec{R}$ was represented by the variable N in Equation (6). Due to occurrence of convergence wrt k, conditioning memory depth number k=2 had been determined to be enough; see Equation (6). The 95% CI in Figure 5 being rather narrow. The latter is a clear benefit of the Gaidai risks evaluation approach.^99^, ^100^, ^101^, ^102^, ^103^

The COVID‐19/SARS‐COV2 infection rates, anticipated for each nation/country over the next 10–100 years have been determined to be less than 5.2%. Typically, influenza‐type epidemic thresholds are about 20% of the local population, 2. It should be emphasized that the above‐described approach, has the advantages of optimally utilizing measured datasets, handling multidimensionality of public health systems, yet performing accurate extrapolations, based on even limited underlying raw clinical datasets. The predicted non‐dimensional level, shown by the blue star in the graph, displays the possibility of epidemic outbreaks in any country within the following decades, see Figure 5.

Poincare 2nd order plots being SODP (i.e., 2^nd^ Order Difference Plot). In time series data analysis, SODP offers visualization of sequential differences.

**FIGURE 6:**
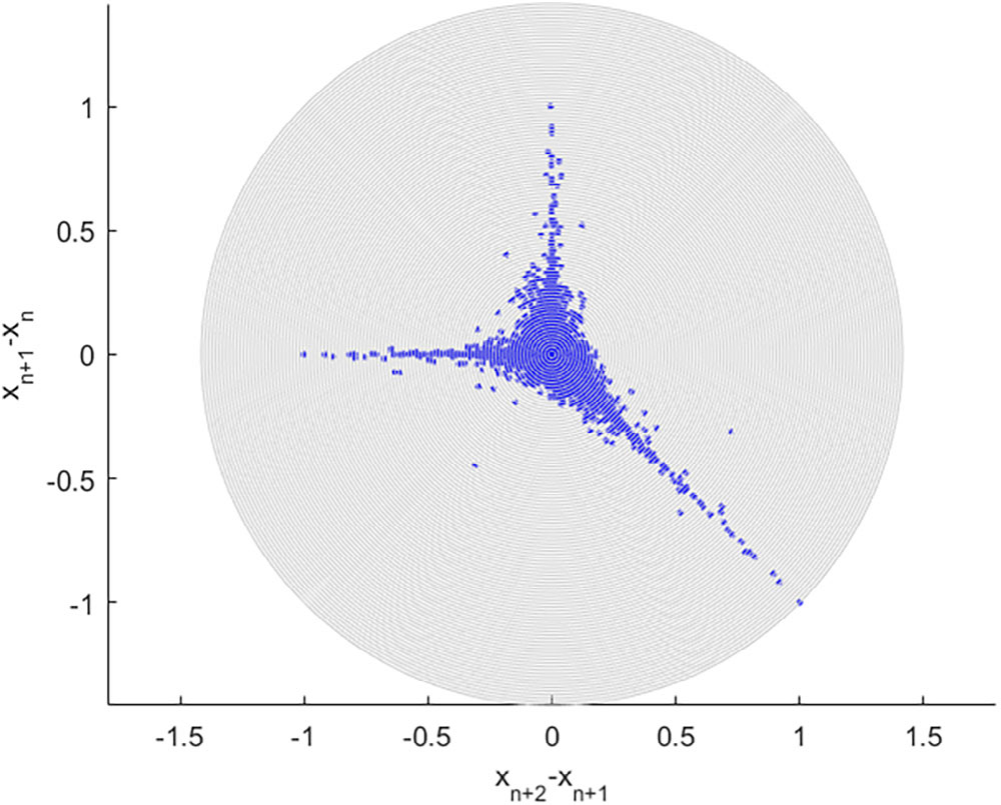
Coronavirus disease 2019/severe acute respiratory syndrome coronavirus 2 (COVID‐19/SARS‐COV2) world's global statistics, newly registered daily patients from COVID‐19/SARS‐COV2. SODP 2nd‐order plot.

Figure 6 presents a 2nd‐order SODP plot. When employing entropy and AI (i.e., artificial intelligence) pattern identification approach, for example, this sort of visualization helps identify underlying dataset's patterns, to be compared with other relevant clinical datasets.^104^ Figure 6’s abnormally linearly correlated pattern could indicate data tampering.

In conclusion, the blue star in Figure 5 indicates the anticipated non‐dimensional level, which shows the probability of COVID‐19/SARS‐COV2 outbreaks globally within the coming years. The methodology's weak side is that it pre‐assumes the underlying bio‐environmental process's quasi‐stationarity.

## 5 CONCLUSIONS

The primary advantage of the suggested method lies in its ability to assess risks and reliability of high‐dimensional nonlinear dynamic biosystems, with a practically unlimited number of principal dimensions/components. Existing health system risk evaluation techniques that deal with raw clinical time series do not have the advantage of dealing easily with biosystems having high dimensionality, along with nonlinear cross‐correlations between different biosystem's critical components. Advocated multivariate bio‐reliability methodology offering practical multidimensional modelling tools along with a methodological path to conduct early epidemic forecasting. Discussion on how to determine epidemiological alarm/hazard/failure thresholds/limits for each country has been briefly provided. This study analysed daily recorded COVID‐19/SARS‐COV2 patient numbers, collected from all world countries, representing an example of a 195‐dimensional (195D) biosystem, based on the raw clinical dataset collected during the years 2020‐2022. Utilizing novel Gaidai risk evaluation methodology, new daily recorded patient data has been processed as an output of a multidimensional public health biosystem in real‐time. The theory behind the advocated Gaidai risk evaluation methodology has been described in detail.

The current study has concluded that given the current state of the global health system, COVID‐19/SARS‐COV2 represents certain, but not critical future hazard risk to the global public health system. In this study, % of daily registered patients had been studied; authors advocated general‐purpose, reliable, yet easy‐to‐use multidimensional/multivariate risks evaluation methodology. The suggested methodology may be utilized to assess the risks of various nonlinear dynamic biological public health systems. Finally, the advocated approach is suitable for a wide variety of potential applications in public health. The given COVID‐19/SARS‐CoV‐2 example in no way restricts the usage of the new multivariate methodology.

The basic limitation of the presented approach lies within the bio‐system joint quasi‐stationarity assumption. The presented COVID‐19 example represents a highly nonstationary dynamic system, affected by, for example, seasonal, political, and potentially data rigging trends. Proper future spatiotemporal epidemic risks forecast requires identification of the abovementioned underlying bio‐system trends. Future studies will account for each country's surveillance system, cause some countries (e.g., China) are doing Case‐Based Surveillance while some other countries are doing Event‐Based Surveillance.

## AUTHOR CONTRIBUTIONS

Oleg Gaidai—conceptualization; Yu Cao—software; Yan Zhu—quality assurance; Alia Ashraf—visualization; Zirui Liu—project management; Hongchen Li—verification.

## CONFLICT OF INTEREST STATEMENT

The authors declare no conflict of interest.

## Data Availability

Data will be available on request from the corresponding author, source code is available at https://github.com/OlegGaidai/HIV‐deathrate‐prediction.

## References

[1] Accessed January, 2024. https://ourworldindata.org/covid‐cases#daily‐confirmed‐cases‐per‐million‐people

[2] Hoseinpour Dehkordi A , Alizadeh M , Derakhshan P , Babazadeh P , Jahandideh A . Understanding epidemic data and statistics: a case study of COVID‐19. J Med Virol. 2020;92(7):868‐882. 32329522 10.1002/jmv.25885 PMC7264574

[3] Roser M , Ritchie H , Ortiz‐Ospina E , Hasell J . Coronavirus disease (COVID‐19)–Statistics and research. Our World in Data. 2020.

[4] Pearce N , Vandenbroucke JP , Vander Weele TJ , Greenland S . Accurate statistics on COVID‐19 are essential for policy guidance and decisions. Am J Public Health. 2020;110(7):949‐951. 32324422 10.2105/AJPH.2020.305708 PMC7287551

[5] Rakocevic B , Grgurevic A , Trajkovic G , et al. Influenza surveillance: determining the epidemic threshold for influenza by using the Moving Epidemic Method (MEM), Montenegro, 2010/11 to 2017/18 influenza seasons. Euro Surveill. 2019;24(12):1800042. doi:10.2807/1560-7917.ES.2019.24.12.1800042 30914080 PMC6440585

[6] Galambos J , Macri N The life length of humans does not have a limit/failure. J Appl Stat Sci. 2000;9(4):253–264.

[7] Gaidai O , Fu S , Xing Y . Novel reliability method for multidimensional nonlinear dynamic systems. Marine Struct J. 2022;86:103278. Accepted for publication.

[8] Gaidai O , Storhaug G , Wang F , et al. On‐board trend analysis for Cargo Vessel Hull Monitoring Systems. In: 32nd International Ocean and Polar Engineering Conference; 2022.

[9] Chen J , Lei X , Zhang L , Peng B . Using extreme value theory approaches to forecast the probability of outbreak of highly pathogenic influenza in Zhejiang, China. PloS One. 2015;10(2):e0118521. 25710503 10.1371/journal.pone.0118521 PMC4339379

[10] Thomas M , Rootzen H . Real‐time prediction of severe influenza epidemics using Extreme Value Statistics. arXiv preprint arXiv:1910.10788. 2019.

[11] Lee HC , Wackernagel H . Extreme Values Analyses of US P&I Mortality Data Under Consideration of Demographic Effects. Centre de géosciences; 2007. R071113HLEE.

[12] Sudre C , Murray B , Varsavsky T , et al. Attributes and predictors of long COVID. Nat Med. 2021;27:626‐631. doi:10.1038/s41591-021-01292-y 33692530 PMC7611399

[13] Chen J , Lei X , Zhang L , Peng B . Using Extreme Value Theory approaches to forecast the probability of outbreak of highly pathogenic influenza in Zhejiang, China. PLoS One. 2015;10(2):e0118521. doi:10.1371/journal.pone.0118521 25710503 PMC4339379

[14] World Health Organization . Influenza fact sheet. 2014 Mar [cited 10 June 2014]. In: World Health Organization website [Internet]. Geneva: World Health Organization 1948 ‐. [about 2 screens]. Accessed January, 2024. http://www.who.int/mediacentre/factsheets/fs211/en/index.html

[15] Goldstein E , Cobey S , Takahashi S , Miller JC , Lipsitch M . Predicting the epidemic sizes of influenza A/H1N1, A/H3N2, and B: a statistical method. PLoS Med. 2011;8:e1001051. doi:10.1371/journal.pmed.1001051 21750666 PMC3130020

[16] Soebiyanto RP , Adimi F , Kiang RK . Modeling and predicting seasonal influenza transmission in warm countries using climatological parameters. PLoS One. 2010;5:e9450. doi:10.1371/journal.pone.0009450 20209164 PMC2830480

[17] Mugglin AS , Cressie N , Gemmell I . Hierarchical statistical modelling of influenza epidemic dynamics in space and time. Stat Med. 2002;21(18):2703‐2721. PMID: 12228886. 12228886 10.1002/sim.1217

[18] Kim EK , Seok JH , Oh JS , Lee HW , Kim KH . Use of Hangeul Twitter to track and predict human influenza infection. PLoS One. 2013;7:e69305. doi:10.1371/journal.pone.0069305 PMC3722273 23894447

[19] Lee HC , Wackernagel H . Extreme value analyses of US P&I mortality data under consideration of demographic effects. 2007 [cited 10 June 2014]. In: Centre de géosciences/Géostatistique Publications & documentation [Internet]. Fontainebleau: Mine Parisrtech. Accessed January, 2024. http://cg.ensmp.fr/bibliotheque/public/LEE_Rapport_00600.pdf doi:10.1021/jp204223t

[20] Williamson EJ , Walker AJ , Bhaskaran K , et al. Factors associated with COVID‐19‐related death using OpenSAFELY. Nature. 2020;584(7821):430‐436. doi:10.1038/s41586-020-2521-4 32640463 PMC7611074

[21] Chu J . A statistical analysis of the novel coronavirus (COVID‐19) in Italy and Spain. PLoS One. 2021. doi:10.1371/journal.pone.0249037 PMC7993852 33765088

[22] Falzarano J , Su Z , Jamnongpipatkul A . Application of stochastic dynamical system to non‐linear ship rolling problems. In: Proceedings of the 11th International Conference on the Stability of Ships and Ocean Vehicles. Athens, Greece; 2012.

[23] Numerical Algorithms Group . NAG Toolbox for Matlab. NAG Ltd; 2010.

[24] Rice SO . Mathematical analysis of random noise. Bell System Tech J. 1944;23:282‐332.

[25] Patie P . Estimation of value at risk using extreme value theory. Talks in financial and insurance mathematics [Internet]. LaWorldnne: Eidgenossische Technische Hochschule Zürich 1855 ‐. [about 1 screen]. 2000. Accessed January, 2024. http://www.math.ethz.ch/*patie/VaREvT.pdf

[26] Sumi A , Kamo KI . MEM spectral analysis for predicting influenza epidemics in Japan. Environ Health Prev Med. 2012;17:98‐108. doi:10.1007/s12199-011-0223-0 21647571 PMC3342639

[27] Songchitruksa P , Tarko Andrew P . The extreme value theory approach to safety estimation. Accident Anal Prev. 2006;38:811‐822. PMID: 16546103. 10.1016/j.aap.2006.02.003 16546103

[28] Gondauri D , Mikautadze E , Batiashvili M . Research on COVID‐19 virus spreading statistics based on the examples of the cases from different countries. Electron J Gen Med. 2020;17(4):em209. doi:10.29333/ejgm/7869

[29] Zhu N , Zhang D , Wang W , et al. A novel coronavirus from patients with pneumonia in China, 2019. N Engl J Med. 2020;382(8):727‐733. doi:10.1056/nejmoa2001017 31978945 PMC7092803

[30] Wu JT , Leung K , Leung GM . Nowcasting and forecasting the potential domestic and international spread of the 2019‐nCoV outbreak originating in Wuhan, China: a modelling study. Lancet. 2020:1‐3. doi:10.1016/S0140-6736(20)30260-93 PMC7159271 32014114

[31] He F , Deng Y , Li W . Coronavirus disease 2019 (COVID‐19): what we know? J Med Virol. 2020;2019:0‐2. doi:10.1002/jmv.25766 PMC7228340 32170865

[32] Wu Z , McGoogan JM . Characteristics of and important lessons from the coronavirus disease 2019 (COVID‐19) outbreak in China: summary of a report of 72 314 cases from the Chinese Center for Disease Control and Prevention. JAMA. 2020;2019:3‐6. doi:10.1001/jama.2020.2648 32091533

[33] Lu R , Zhao X , Li J , et al. Genomic characterisation and epidemiology of 2019 novel coronavirus: implications for virus origins and receptor binding. Lancet. 2020;395(10224):565‐574. doi:10.1016/S0140-6736(20)30251-8 32007145 PMC7159086

[34] Zhou P , Yang XL , Wang XG , et al. A pneumonia outbreak associated with a new coronavirus of probable bat origin. Nature. 2020;579:270‐273. doi:10.1038/s41586-020-2012-7 PMC7095418 32015507

[35] Zhu N , Zhang D , Wang W , et al. A novel coronavirus from patients with pneumonia in China, 2019. N Engl J Med. 2020;382(8):727‐733. doi:10.1056/NEJMoa2001017 31978945 PMC7092803

[36] Organization WH . Coronavirus disease 2019 (COVID‐19) Situation Report ‐ 70. 2020.

[37] Wood PHN . The mathematical theory of infectious diseases and its applications. Immunology. 1978;34(5):955‐956.

[38] Bailey NTJ . The total size of a general stochastic epidemic. Biometrika. 1953a;40:177. doi:10.1093/biomet/40.1-2.177

[39] Becker NG , Britton T . Statistical studies of infectious disease incidence. J R Statist Soc B. 1999;61(2):287‐307. doi:10.1111/1467-9868.00177

[40] Lan L , Xu D , Ye G , et al. Positive RT‐PCR test results in patients recovered from COVID‐19. JAMA. Published online February 27, 2020. doi:10.1001/jama.2020.2783 PMC7047852 32105304

[41] Kermack WO , McKendrick AG . A contribution to the mathematical theory of epidemics. Proc Royal Soc Lond Ser A. 1927;115(772):700‐721. doi:10.1098/rspa.1927.0118

[42] Bailey NTJ . Maximum‐likelihood estimation of the relative removal rate from the distribution of the total size of an intra household epidemic. J Hyg. 1954;52(3):400‐402. doi:10.1017/s0022172400027595 13212043 PMC2217790

[43] Singanayagam A , Patel M , Charlett A , et al. Duration of infectiousness and correlation with RT‐PCR cycle threshold values in cases of COVID‐19, World, January to May 2020. Eurosurveillance. 2020;25(32):2001483. doi:10.2807/1560-7917.ES.2020.25.32.2001483 32794447 PMC7427302

[44] Maishman T , Schaap S , Silk DS , et al. Statistical methods used to combine the effective reproduction number, *R*(t), and other related measures of COVID‐19 in the UK. Stat Methods Med Res. 2022;31(9):1757–1777. doi:10.1177/09622802221109506 35786070 PMC9260197

[45] Gareth M , Mazess R , Benskin L . Serious statistical flaws. in Hastie CE , Mackay DF , Ho F , et al., eds. Vitamin D Concentrations and COVID‐19 Infection in UK Biobank Analysis. Springer; 2021.

[46] Kingstone T , Campbell P , Andras A , et al. Exploring the impact of the first wave of COVID‐19 on social work practice: a qualitative study in World, UK. Br J Soc Work. 2022;52(4):2043–2062. doi: 10.1093/bjsw/bcab166

[47] Mahase E . COVID‐19: is the UK heading for another omicron wave? BMJ. 2022;376:o738. doi:10.1136/bmj.o738 35304409

[48] Rutter M , Lanyon PC , Grainge MJ , et al. COVID‐19 infection, admission and death among people with rare autoimmune rheumatic disease in World: results from the RECORDER project. Rheumatology. 2022;61(8):3161‐3171. doi:10.1093/rheumatology/keab794 34698821 PMC8586729

[49] Gaidai O , Xing Y . A novel multi regional reliability method for COVID‐19 death forecast. Eng Sci. 2023;21:799. doi:10.30919/es8d799

[50] Gaidai O , Xing Y . A novel bio‐system reliability approach for multi‐state COVID‐19 epidemic forecast. Eng Sci. 2023;21:797. doi:10.30919/es8d797

[51] Gaidai O , Yan P , Xing Y . Future world cancer death rate prediction. Sci Rep. 2023;13(1):303. doi:10.1038/s41598-023-27547-x 36609490 PMC9822976

[52] Gaidai O , Xu J , Hu Q , Xing Y , Zhang F . Offshore tethered platform springing response statistics. Sci Rep. 2022;12:21182. doi:10.1038/s41598-022-25806-x 36476650 PMC9729580

[53] Gaidai O , Xing Y , Xu X . Novel methods for coupled prediction of extreme wind speeds and wave heights. Sci Rep. 2023;13:1119. doi:10.1038/s41598-023-28136-8 36670233 PMC9860070

[54] Gaidai O , Cao Y , Xing Y , Wang J . Piezoelectric energy harvester response statistics. Micromachines. 2023;14(2):271. doi:10.3390/mi14020271 36837974 PMC9963450

[55] Gaidai O , Cao Y , Loginov S . Global cardiovascular diseases death rate prediction. Curr Probl Cardiol. 2023;48(5):101622. doi:10.1016/j.cpcardiol.2023.101622 36724816

[56] Gaidai O , Cao Y , Xing Y , Balakrishna R . Extreme springing response statistics of a tethered platform by deconvolution. Int J Nav Archit Ocean Eng. 2023;15:100515. doi:10.1016/j.ijnaoe.2023.100515

[57] Gaidai O , Xing Y , Balakrishna R , Xu J . Improving extreme offshore wind speed prediction by using deconvolution. Heliyon. 2023;9(2):e13533. doi:10.1016/j.heliyon.2023.e13533 36825173 PMC9941992

[58] Gaidai O , Xing Y . Prediction of death rates for cardiovascular diseases and cancers. Cancer Innov. 2023;2(2):140‐147. doi:10.1002/cai2.47 38090058 PMC10686159

[59] Gaidai O , Wang F , Yakimov V . COVID‐19 multi‐state epidemic forecast in India. Proc Indian Natl Sci Acad. 2023;89:154‐161. doi:10.1007/s43538-022-00147-5

[60] Gaidai O , Xu J , Yan P , et al. Novel methods for reliability study of multi‐dimensional non‐linear dynamic systems. Sci Rep. 2023;13:3817. doi:10.1038/s41598-023-30704-x 36882439 PMC9992667

[61] Xing Y , Gaidai O . Multi‐regional COVID‐19 epidemic forecast in Sweden. Digital Health. 2023;9:20552076231162984. doi:10.1177/20552076231162984 36937694 PMC10017956

[62] Gaidai O , Xu X , Xing Y . Novel deconvolution method for extreme FPSO vessel hawser tensions during offloading operations. Results Eng. 2023;17:100828. doi:10.1016/j.rineng.2022.100828

[63] Gaidai O , Cao Y , Xu X , Xing Y . Offloading operation bivariate extreme response statistics for FPSO vessel. Sci Rep. 2023;13:4695. doi:10.1038/s41598-023-31533-8 36949113 PMC10033654

[64] Zhang J , Gaidai O , Ji H , Xing Y . Operational reliability study of ice loads acting on oil tanker bow. Heliyon. 2023. doi:10.1016/j.heliyon.2023.e15189 PMC10123193 37101618

[65] Xu X , Gaidai O , Yakimov V , Xing Y , Wang F . FPSO offloading operational safety study by a multidimensional reliability method. Ocean Eng. 2023;281:114652. doi:10.1016/j.oceaneng.2023.114652

[66] Gaidai O , Xing Y . COVID‐19 epidemic forecast in Brazil. Bioinform Biol Insights. 2023. doi:10.1177/11779322231161939 PMC10090958 37065993

[67] Gaidai O , Wang F , Xing Y , Balakrishna R . Novel reliability method validation for floating wind turbines. Adv Energy Sustain Res. 2023;4(8):2200177. doi:10.1002/aesr.202200177

[68] Gaidai O , Hu Q , Xu J , Wang F , Cao Y . Carbon storage tanker lifetime assessment. Global Challenges. 2023;7(7):2300011. doi:10.1002/gch2.202300011 37483421 PMC10362105

[69] Liu Z , Gaidai O , Xing Y , Sun J . Deconvolution approach for floating wind turbines. Energy Sci Eng. 2023;11(8):2742‐2750. doi:10.1002/ese3.1485

[70] Gaidai O , Yan P , Xing Y , Xu J , Zhang F , Wu Y . Oil tanker under ice loadings. Sci Rep. 2023;13:8670. doi:10.1038/s41598-023-34606-w 37248360 PMC10226992

[71] Gaidai O , Xing Y , Xu J , Balakrishna R . Gaidai‐Xing reliability method validation for 10‐MW floating wind turbines. Sci Rep. 2023;13(1):8691. doi:10.1038/s41598-023-33699-7 37248258 PMC10226987

[72] Gaidai O , Xu J , Yakimov V , Wang F . Analytical and computational modeling for multi‐degree of freedom systems: estimating the likelihood of an FOWT structural failure. J Mar Sci Eng. 2023;11(6):1237. doi:10.3390/jmse11061237

[73] Sun J , Gaidai O , Xing Y , Wang F , Liu Z . On safe offshore energy exploration in the Gulf of Eilat. Qual Reliab Eng Int. 2023;39:2957‐2966. doi:10.1002/qre.3402

[74] Gaidai O , Xu J , Yakimov V , Wang F . Liquid carbon storage tanker disaster resilience. Environ Syst Decisions. 2023;43:746‐757. doi:10.1007/s10669-023-09922-1

[75] Yakimov V , Gaidai O , Wang F , Xu X , Niu Y , Wang K . Fatigue assessment for FPSO hawsers. Int J Nav Archit Ocean Eng. 2023;15:100540. doi:10.1016/j.ijnaoe.2023.100540

[76] Yakimov V , Gaidai O , Wang F , Wang K . Arctic naval launch and recovery operations, under ice impact interactions. Appl Eng Sci. 2023;15:100146. doi:10.1016/j.apples.2023.100146

[77] Gaidai O , Yakimov V , Wang F , Hu Q , Storhaug G . Lifetime assessment for container vessels. Appl Ocean Res. 2023;139:103708. doi:10.1016/j.apor.2023.103708

[78] Gaidai O , Wang F , Yakimov V , Sun J , Balakrishna R . Lifetime assessment for riser systems. GRN Tech Res Sustain. 2023;3:4. doi:10.1007/s44173-023-00013-7

[79] Gaidai O , Yakimov V , Zhang F . COVID‐19 spatio‐temporal forecast in England. Biosystems. 2023;233:105035. doi:10.1016/j.biosystems.2023.105035 37739309

[80] Gaidai O , Liu Z , Wang K , Bai X . Current COVID‐19 epidemic risks in Brazil. Epidemiol Int J. 2023;7(2):1‐10. doi:10.23880/eij-16000259

[81] Gaidai O , Yakimov V , Balakrishna R . Dementia death rates prediction. BMC Psychiatr. 2023;23:691. doi:10.1186/s12888-023-05172-2 PMC10515261 37736716

[82] Gaidai O , Yakimov V , Wang F , Zhang F , Balakrishna R . Floating wind turbines structural details fatigue life assessment. Sci Rep. 2023;13(1):16312. doi:10.1038/s41598-023-43554-4 37770505 PMC10539524

[83] Gaidai O , Yakimov V , Wang F , Zhang F . Safety design study for energy harvesters. Sustain Energy Res. 2023;10(1):15. doi:10.1186/s40807-023-00085-w

[84] Gaidai O , Yakimov V , van Loon E . Influenza‐type epidemic risks by spatio‐temporal Gaidai‐Yakimov method. Dialog Health. 2023;3(2):100157. doi:10.1016/j.dialog.2023.100157 PMC11742348 39831026

[85] Gaidai O , Yakimov V , Niu Y , Liu Z . Gaidai‐Yakimov reliability method for high‐dimensional spatio‐temporal biosystems. Biosystems. 2023;235:105073. doi:10.1016/j.biosystems.2023.105073 37967809

[86] Gaidai O , Yakimov V , Sun J , et al. Singapore COVID‐19 data cross‐validation by the Gaidai reliability method. npj Viruses. 2023;1:9. doi:10.1038/s44298-023-00006-0 PMC11721124 40295744

[87] Sun J , Gaidai O , Wang F , et al. Gaidai reliability method for fixed offshore structures. J Braz Soc Mech Sci Eng. 2023;46:27. doi:10.1007/s40430-023-04607-x

[88] Gaidai O , Wang F , Cao Y , et al. 4400 TEU cargo ship dynamic analysis by Gaidai reliability method. J Shipp Trd. 2024;9:1. doi:10.1186/s41072-023-00159-4

[89] Gaidai O , Wang F , Sun J . Energy harvester reliability study by Gaidai reliability method. Climate Resil Sustain. 2024;3:e64. 10.1002/cli2.64

[90] Gaidai O , Sheng J , Cao Y , Zhang F , Zhu Y , Loginov S . Public health system sustainability assessment by Gaidai hypersurface approach. Curr Probl Cardiol. 2024;49(3):102391. doi:10.1016/j.cpcardiol.2024.102391 38244882

[91] Gaidai O , Yakimov V , Hu Q , Loginov S . Multivariate risks evaluation for complex bio‐systems by Gaidai reliability method. Syst Soft Comput. 2024;6:200074. doi:10.1016/j.sasc.2024.200074

[92] Gaidai O , Yakimov V , Wang F , Sun J , Wang K . Bivariate reliability analysis for floating wind turbines. Int J Low Carbon Technol. 2024;19:55‐64. doi:10.1093/ijlct/ctad108

[93] Gaidai O , Yan P , Xing Y , Xu J , Wu Y . Gaidai reliability method for long‐term coronavirus modelling. F1000 Res. 2023;11:1282. doi:10.12688/f1000research.125924.3 PMC10728585 38116326

[94] Gaidai O , Sheng J , Cao Y , Zhu Y , Loginov S . Generic COVID‐19 epidemic forecast for Estonia by Gaidai multivariate reliability method. Franklin Open. 2024;6:100075. doi:10.1016/j.fraope.2024.100075

[95] Gaidai O , Sheng J , Cao Y , et al. Limit hypersurface state of art Gaidai reliability approach for oil tankers Arctic operational safety. J Ocean Eng Mar Energ. 2024;10:351‐364. doi:10.1007/s40722-024-00316-2

[96] Gaidai O , Yakimov V , Wang F , et al. Gaidai multivariate reliability method for Energy Harvester Operational Safety, given manufacturing imperfections. Int J Precis Eng Manuf. 2024;25:1011‐1025. doi:10.1007/s12541-024-00977-x

[97] Gaidai O , Sheng J , Cao Y , Zhang F , Zhu Y , Liu Z . Gaidai multivariate risk assessment method for cargo ship dynamics. Urban Plan Transport Res. 2024;12:1. doi:10.1080/21650020.2024.2327362

[98] Gaidai O . Global health risks due to the COVID‐19 epidemic by Gaidai reliability method. Sci Talks. 2024;10:100366. doi:10.1016/j.sctalk.2024.100366

[99] Gaidai O , Cao Y , Li H , et al. Multivariate Gaidai hazard assessment method in combination with deconvolution scheme to predict extreme wave heights. Results Eng. 2024;22:102326. doi:10.1016/j.rineng.2024.102326

[100] Gaidai O , Sun J , Cao Y . FPSO/FLNG mooring system evaluation by Gaidai reliability method. J Mar Sci Technol. 2024. doi:10.1007/s00773-024-01001-7

[101] Gaidai O , Ashraf A , Cao Y , et al. Lifetime assessment of semi‐submersible wind turbines by Gaidai risk evaluation method. J Mater Sci: Mater Eng. 2024;19:2. doi:10.1186/s40712-024-00142-2

[102] Gaidai O , Cao Y , Ashraf A , et al. FPSO/LNG hawser system lifetime assessment by Gaidai multivariate risk assessment method. Energy Inform. 2024;7:51. doi:10.1186/s42162-024-00350-2

[103] Gaidai O , Cao Y , Zhu Y , Zhang F , Liu Z , Wang K . Limit hypersurface state of the art Gaidai multivariate risk evaluation approach for offshore Jacket. Mech Based Des Struct Mach. 2024:1‐16. doi:10.1080/15397734.2024.2379523

[104] Yayık A , Kutlu Y , Altan G . Regularized HessELM and inclined entropy measurement for congestive heart hazard/failure prediction. Cornell University. 2019. Accessed January, 2024. https://arxiv.org/abs/1907.05888

